# Metabolic consequences of microRNA-122 inhibition in rainbow trout, *Oncorhynchus mykiss*

**DOI:** 10.1186/1471-2164-15-70

**Published:** 2014-01-27

**Authors:** Jan A Mennigen, Christopher J Martyniuk, Iban Seiliez, Stéphane Panserat, Sandrine Skiba-Cassy

**Affiliations:** 1 Institut National de la Recherche Agronomique (INRA), Nutrition, Metabolism and Aquaculture Unit (UR1067), Saint-Pée-sur-Nivelle F-64310, France; 2 Canadian Rivers Institute and Department of Biology, University of New Brunswick, Saint John, NB E2L 4 L5, Canada

**Keywords:** Glycolysis, Lipogenesis, MicroRNA, Fish, Metabolism, Gene expression, Insulin signaling

## Abstract

**Background:**

MicroRNAs (miRNAs) are small regulatory molecules which post-transcriptionally regulate mRNA stability and translation. Several microRNAs have received attention due to their role as key metabolic regulators. In spite of the high evolutionary conservation of several miRNAs, the role of miRNAs in lower taxa of vertebrates has not been studied with regard to metabolism. The liver-specific and highly abundant *miRNA-122* is one of the most widely studied miRNA in mammals, where it has been implicated in the control of hepatic lipid metabolism. Following our identification of acute postprandial, nutritional and endocrine regulation of hepatic *miRNA-122* isomiRNA expression in rainbow trout, we used complementary *in silico* and *in vivo* approaches to study the role of *miRNA-122* in rainbow trout metabolism. We hypothesized that the role of *miRNA-122* in regulating lipid metabolism in rainbow trout is conserved to that in mammals and that modulation of *miRNA-122* function would result in altered lipid homeostasis and secondarily altered glucose homeostasis, since lipogenesis has been suggested to act as glucose sink in trout.

**Results:**

Our results show that *miRNA-122* was functionally inhibited *in vivo* in the liver. Postprandial glucose concentrations increased significantly in rainbow trout injected with a *miRNA-122* inhibitor, and this effect correlated with decreases in hepatic FAS protein abundance, indicative of altered lipogenic potential. Additionally, *miRNA-122* inhibition resulted in a 20% decrease in plasma cholesterol concentration, an effect associated with increased expression of genes involved in cholesterol degradation and excretion.

**Conclusions:**

Overall evidence suggests that *miRNA-122* may have evolved in early vertebrates to support liver-specific metabolic functions. Nevertheless, our data also indicate that metabolic consequences of *miRNA-122* inhibition may differ quantitatively between vertebrate species and that distinct direct molecular targets of *miRNA-122* may mediate metabolic effects between vertebrate species, indicating that *miRNA-122* - mRNA target relationships may have undergone species-specific evolutionary changes.

## Background

MicroRNAs (miRNAs) are a small class of non-coding RNAs, which were first described in *Caenorhabditis elegans*[1]. The class of miRNAs are posttranscriptional regulators that mediate their physiological effects both by target mRNA degradation and translational inhibition [2]. Following their discovery, miRNAs have been identified in all vertebrate classes [3], and some miRNAs have been found to be expressed in a tissue-specific manner [4]. In the mammalian liver, the miRNA expression profile is dominated by a single sequence, miR-122, with approximately 50 000 copies per cell, representing close to 70% of the overall miRNAs species in this tissue [4]. In mammals, *miRNA-122* has been shown to be implicated in the differentiation and maintenance of the hepatic phenotype [5-8], and the regulation of metabolic liver functions, especially lipid metabolism. Specifically, *miRNA-122* exerts a stimulatory role in lipogenesis and cholesterol synthesis on the one hand, as well as an inhibitory effect on β-oxidation capacity on the other [9-11]. The tissue specific expression and high abundance of *miRNA-122*, as well as the comparatively large amount of mammalian data which made *miRNA-122* a ‘paradigm’ in miRNA research [12], make this miRNA a particularly suitable target of study for comparative investigation. In rainbow trout (*Oncorhynchus mykiss*), *miRNA-122* is specifically localized to the liver [13], where it is highly abundant [14], reflecting the situation in mammals. We have previously shown that a specific isoform of *miRNA-122*, *omy-miRNA-122b,* is postprandially regulated in rainbow trout [14], and that this regulation depends on macronutrient composition and the endocrine factor insulin [15], a key metabolic hormone. These findings led us to investigate the function of *miRNA-122* in rainbow trout, which, in spite of its high degree of evolutionary conservation [16], has not been characterized functionally in lower vertebrates. Specifically, given its key implication in lipid metabolism in higher vertebrates, we investigated the hypothesis, that *miRNA-122* is equally involved in the regulation of lipid metabolism in trout, and that, via modulation of lipid metabolism, it may secondarily regulate postprandial glucose metabolism in this species. Rainbow trout are carnivorous fish which are considered poor utilizers of dietary carbohydrate [17], but several lines of evidence have shown that induction of hepatic *de novo* lipogenesis may act as a ‘glucose-sink’ in rainbow trout, resulting in improved utilization of glucose. For example an improved postprandial glucose profile was observed in a ‘fat line’ of rainbow trout, characterized by increased expression of genes involved in *de novo* lipogenesis in the liver, as well as increased muscle fat content [18,19]. Pharmacological modulation of rainbow trout by the antidiabetic drug metformin equally resulted in improved postprandial glucose clearance, but in contrast to mammals, this effect was not correlated with a repression of hepatic gluconeogenic gene expression, but rather with an induction of hepatic lipogenic gene expression [20], providing further evidence for an involvement of hepatic *de novo* lipogenesis in glucose clearance in rainbow trout. Acute administration of insulin in rainbow trout resulted in increased *fas* gene expression, protein abundance and enzymatic activity of FAS [21], and this acute, insulin-stimulated FAS activity has been shown to increase hepatic triglyceride synthesis [22], which may, in part, contribute to improved glucose utilization induced by insulin [21]. Lastly, a link between hepatic glucose utilization and lipogenesis has also been shown at the metabolite level in rainbow trout, as radioactively labeled glucose is dose-dependently metabolized into triglycerides [23,24]. In order to investigate a possible role for *miRNA-122* in coordinating postprandial glucose homeostasis through the modulation of lipid metabolism, we used an *in vivo* approach by blocking the activity of *miRNA-122* using a short LNA-*miRNA-122* inhibitor (LNA-122i) [25]. This approach was complemented by *in silico* approaches to predict potential direct mRNA targets of *miR-122* in rainbow trout, as well as to predict potentially regulated pathways.

## Results

### Mature miRNA-122 is highly conserved in the vertebrate lineage

**Figure 1:**
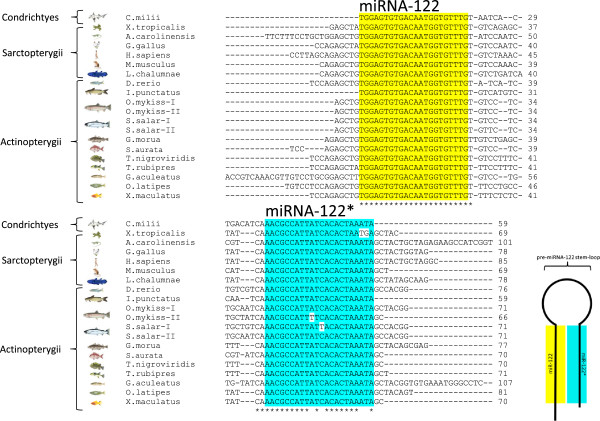
**Evolutionary conservation of *pre-miRNA-122 *in several vertebrate genomes.** Sequences were retrieved from databases indicated in the text and aligned using ClustalW (http://www.ebi.ac.uk/Tools/msa/clustalw2). The complete conservation of the sequence of processed *miRNA-122* (highlighted in yellow) and the high conservation of its complementary strand miRNA-122* (highlighted in blue), is shown, as well as the contribution of each sequence to the structure formed by the *pre-miRNA-122* molecule.

The comparison of genomic sequences between several fish species and higher vertebrates reveals complete evolutionary conservation of the mature *miRNA-122* sequence in vertebrates (Figure 1). The complimentary strand, miRNA-122*, reveals an overall high conservation, however differences in nucleotides do exist among species. The loop region linking both strands does not display a high degree of sequence conservation, in line with its function as a connecting structural component in the hairpin structure (Figure 1). Precursors containing *miRNA-122* sequences are present in the genomes of all vertebrate classes, but are absent from the sea lamprey (*Petromyzon marinus*) genome (data not shown).

### Predicted direct miRNA-122 target genes in rainbow trout are enriched for proliferation and differentiation processes, but not metabolism

**Figure 2:**
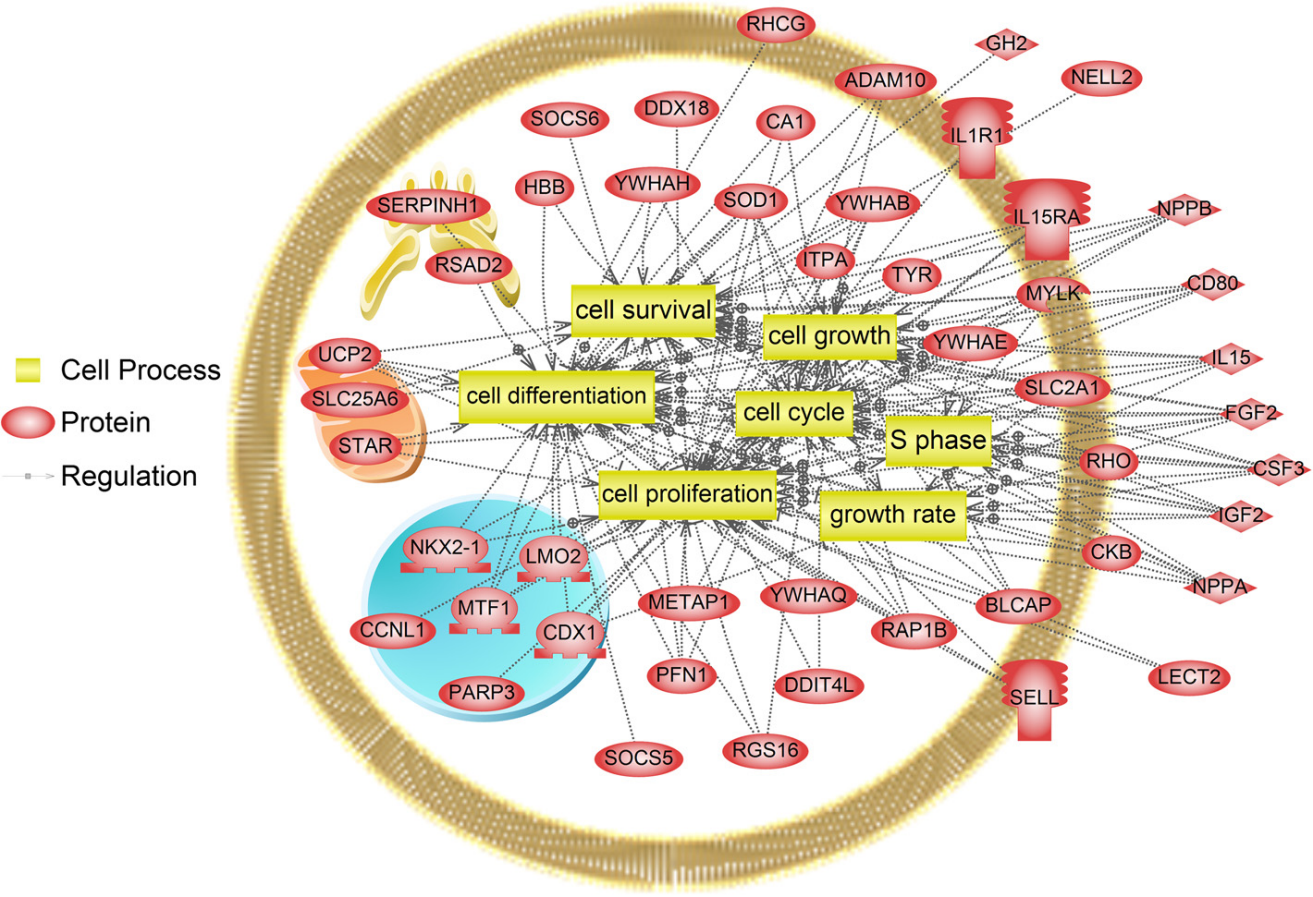
**Rainbow trout targets were identified and their functional enrichment for specific GO terms analyzed as described in the text.** A particular enrichment was noted for pathways involved in cell cycle regulation, cell proliferation and differentiation. Compared to the comprehensive identification and validation of murine targets provided by Tsai et al. [30], 11% of rainbow trout targets were predicted to be conserved targets. All of the conserved targets (*dditl4*, *igf2*, *parp3*, *rtn3*, *serpinh1*, *socs6*, *ucp2a*, *slc2a1* and *adam10*) are enriched in pathways regulating cell cycle, cell proliferation and differentiation, indicating an evolutionary conservation of specific *miRNA-122* target relationships that govern these processes.

The predicted direct *miRNA-122* target genes in rainbow trout (Additional file 1), are highly enriched for cell cycle regulation, as well as cell proliferation and differentiation processes (Figure 2). With the exception of glucose metabolism, no distinct metabolic pathways feature in the list of enriched target genes (SNEA cell process sheet, line 31; Additional file 1).

### Hepatic omy-miRNA-122 isomiRNAs are functionally inhibited in LNA-122i injected rainbow trout

**Figure 3:**
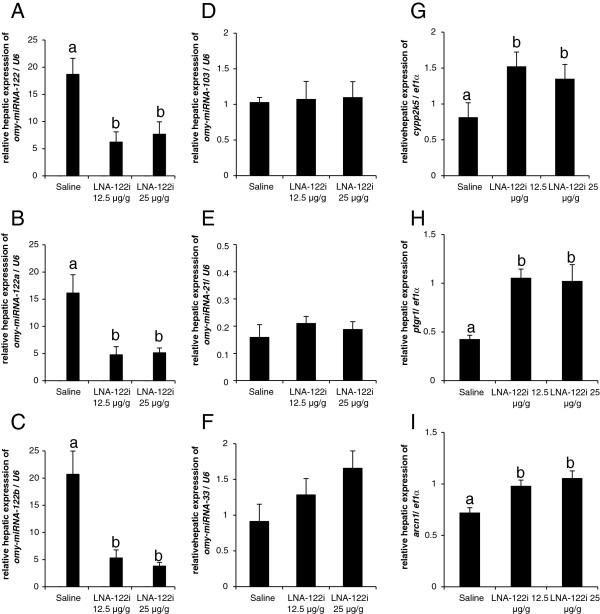
**Effect of LNA-122i treatment on hepatic *omy-miRNA-122 *isomiRNA expression (A-C), expression of hepatic, non-targeted *omy-miRNA *species (D-F) and hepatic mRNAs predicted *in silico *to be targeted by *omy-miRNA-122 *isomiRNAs (G-I).** Analyzed groups consisted of n = 6 samples and data are depicted as mean ± S.E.M. for each group. Data were analyzed using one-way ANOVA and differences between individual experimental groups assessed by Student-Newman-Keuls post-hoc test. Different letters indicate a significant difference (p < 0.05) between experimental groups.

Expression of all hepatic *omy-miRNA-122* isomiRNA sequences, mature *omy-miRNA-122* (df = 2; F = 6.824; p < 0.01), *omy-miRNA-122a* (df = 2; F = 8.708; p < 0.01) and *omy-miRNA-122b* (df = 2; F = 16.9; p < 0.01) was significantly inhibited by LNA-122i treatments, irrespective of dose (Figure 3A-C). Conversely, hepatic expression of *omy-miRNA-103* (df = 2; F = 0.041; p > 0.05), *omy-miRNA-33* (df = 2, F = 1.999; p > 0.05) and *omy-miRNA-21* (df = 2; F = 1.127; p > 0.5) did not change significantly with LNA-122i treatment (Figure 3D-F). The expression of individual *in silico* predicted putative direct *omy-miRNA-122* targets in rainbow trout (Additional file 1) was measured to validate the functionality of *omy-miRNA-122* isomiRNA inhibition (Figure 3G-I). Hepatic expression of fish-lineage specific *cyp2k5* (df = 2; F = 6.53; p < 0.01) increased significantly in fish injected with either dose of LNA-122i (Figure 3G). A significant de-repression of prostaglandin reductase 1, *ptgr1* (df = 2; F = 10.09; p < 0.01; Figure 3H) was observed in fish injected with LNA-122i, as well as an increase in archain1, *arcn1* (df = 2; F = 8.209; p < 0.01; Figure 3I).

### Inhibition of miRNA-122 results in postprandial hyperglycemia and decreased lipid availability

**Figure 4:**
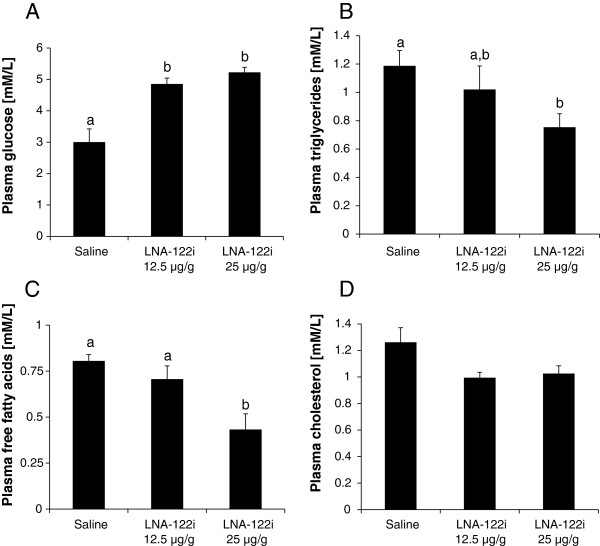
**Effect of LNA-122i treatment on plasma concentrations of the metabolites glucose (A), triglycerides (B), free fatty acids (C) and cholesterol (D).** Analyzed groups consisted of n = 6 samples and data are depicted as mean ± S.E.M. for each group. Data were analyzed using one-way ANOVA and differences between individual experimental groups assessed by Student-Newman-Keuls post-hoc test. Different letters indicate a significant difference (p < 0.05) between experimental groups.

Plasma glucose concentration increased by 62% an 67%, respectively with LNA-122i treatments and this increase was significant (df = 2; F = 17.93; p < 0.01), irrespective of dose (Figure 4A). With regard to lipid metabolites, both triglyceride (−36%) and free fatty acid concentration (−46%) in the plasma decreased in fish treated with the higher dose of 25 μg/g LNA-122i, and both decreases were significant (df = 2; F = 4.017; p < 0.05 and df = 2; F = 7.07; p < 0.01; Figure 4B-C). The LNA-122i treatments lead to small (−20%), but significant decreases in cholesterol (df = 2; F = 3.038, p < 0.05), which, however, did not result in significant pair-wise comparisons between individual treatment groups (Figure 4D).

### Gene expression of hepatic metabolic markers is regulated by miRNA-122 inhibition

#### Effect of omy-miRNA-122 inhibition on genes involved in hepatic glucose metabolism

**Figure 5:**
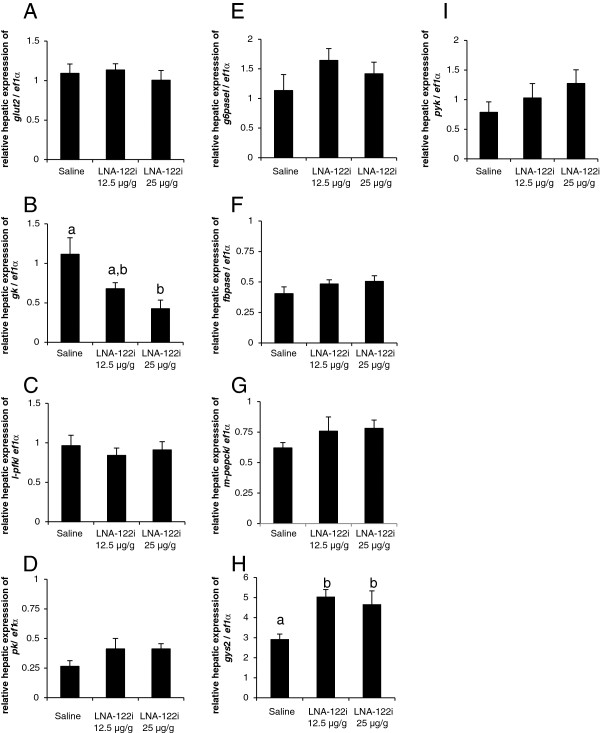
**Effect of LNA-122i treatment on hepatic expression of genes implicated in glucose metabolism.** Expression of genes involved in glucose transport **(A)**, glycolysis **(B-D)**, gluconeogenesis **(E-G)** and glycogen metabolism **(H-I)** was analyzed analyzed using one-way ANOVA and differences between individual experimental groups assessed by Student-Newman-Keuls post-hoc test. Analyzed groups consisted of n = 6 samples and data are depicted as mean ± S.E.M. for each group. Different letters indicate a significant difference (p < 0.05) between experimental groups.

The effect of *miRNA-122* inhibition on the expression of genes involved in hepatic glucose metabolism, specifically glucose import, glycolysis, gluconeogenesis and glycogen metabolism was measured (Figure 5). The expression of the hepatic glucose transporter, *glut2,* did not change significantly with LNA-122i treatment (df = 2; F = 0.15; p > 0.862; Figure 5A). With regard to gene expression of components of the glycolytic pathway, a decrease in expression of glucokinase, *gk*, was observed in fish injected with LNA-122i (df = 2; F = 7.025; p < 0.05; Figure 5B), which was significantly decreased when comparing fish injected with a dose of 25 μg/g LNA-122i to saline-injected control fish. The expression of neither liver phosphofructokinase, *l-pfk* (df = 2; F = 0.774; p > 0.05; Figure 5C), nor pyruvate kinase, *pk* (df = 2; F = 0.774; p > 0.05; Figure 5D) showed any changes between treatment groups.

The expression of the gluconeogenic genes glucose-6-phosphatase 1, *g6pase1* (df = 2; F = 1.303; p > 0.05; Figure 5E), fructose 1,6-bisphophatase, *fbpase* (df = 2; F = 1.345; p > 0.05; Figure 5F), and mitochondrial phospho*enol*pyruvate carboxykinase, *m-pepck* (df = 2; F = 1.371; p > 0.05; Figure 5G), did not change between treatments. The expression of genes involved in glycogen metabolism, glycogen synthase, *gys2* (df = 2; F = 6.01 p < 0.05; Figure 5H) increased in fish treated with either dose of LNA-122i, while glycogen phosphorylase, *pyk*, (df = 2; F = 1.529 p > 0.5; Figure 5I), did not exhibit significant changes between treatment groups.

#### Effect of omy-miRNA-122 inhibition on genes involved in hepatic lipid metabolism

**Figure 6:**
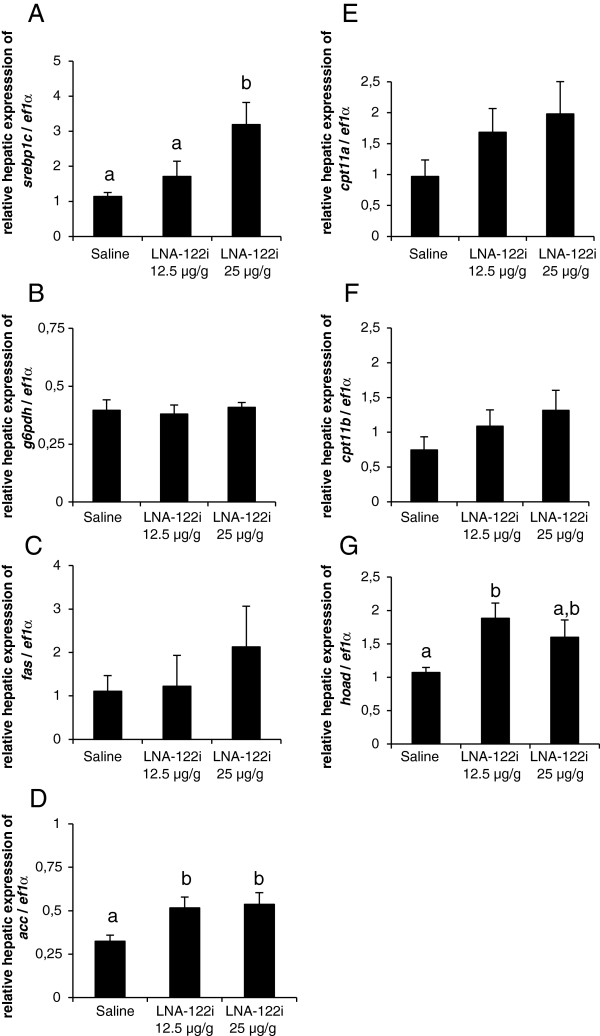
**Effect of LNA-122i treatment on hepatic expression of genes implicated in lipid metabolism.** Expression of genes with roles in lipogenesis **(A-D)** and β-oxidation of fatty acids **(E-G)** was analyzed using one-way ANOVA and differences between individual experimental groups assessed by Student-Newman-Keuls post-hoc test. Analyzed groups consisted of n = 6 samples and data are depicted as mean ± S.E.M. for each group. Different letters indicate a significant difference (p < 0.05) between experimental groups.

The effect of *miRNA-122* inhibition on hepatic expression of genes involved in hepatic lipid metabolism, specifically lipogenesis, and β-oxidation, were analyzed (Figure 6). The expression of sterol regulatory binding protein 1c, *srebp1c* (df = 2; F = 6.176; p < 0.05; Figure 6A), increased significantly compared to saline control in fish injected with 25 μg/g LNA-122i. The expression of genes implicated in lipogenesis did not change between treatments for glucose-6-phosphate dehydrogenase, *g6pdh* (df = 2; F = 0.153; p > 0.05; Figure 6B), or fatty acid synthase, *fas* (df = 2; F = 0.317; p > 0.05; Figure 6C), but the expression of acetyl-CoA-carboxylase, *acc* (df = 2; F = 5.07;p < 0.05; Figure 6D), increased significantly in LNA-122i treated fish, irrespective of the administered dose. With regard to the expression of genes implicated in fatty acid β-oxidation pathways, the expression of carnithine palomtyl transporter isoforms, *cpt1a* (df = 2; F = 1.905; p > 0.05; Figure 6E), and *cpt1b* (df = 2; F = 1.426; p > 0.05; Figure 6F), did not change significantly with treatment, while expression of 3-hydroxyacyl-CoA dehydrogenase, *hoad* (df = 2; F = 3.897; p < 0.05; Figure 6G), was significantly increased in fish injected with 12.5 μg/g LNA-122i compared to saline-injected control fish.

**Figure 7:**
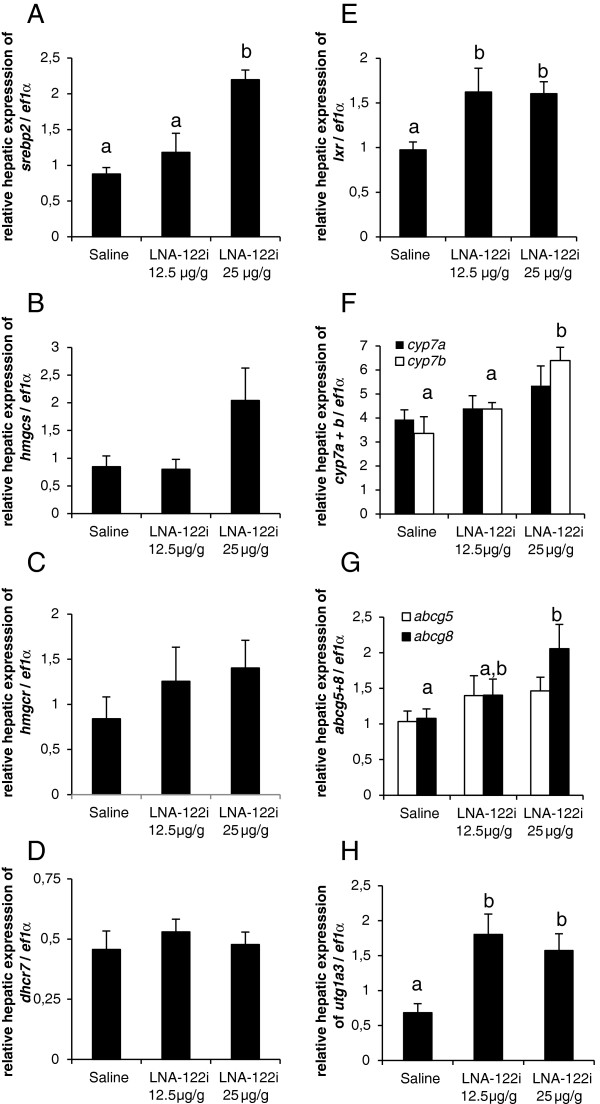
**Effect of LNA-122i treatment on hepatic expression of genes implicated in hepatic cholesterol metabolism.** Expression of genes with roles in cholesterol synthesis **(A-D)**, and cholesterol degradation and excretion **(E-H)**, were analyzed using one-way ANOVA and differences between individual experimental groups assessed by Student-Newman-Keuls post-hoc test. Analyzed groups consisted of n = 6 samples and data are depicted as mean ± S.E.M. for each group. Different letters indicate a significant difference (p < 0.05) between experimental groups.

*Effect of omy-miRNA-122 inhibition on genes involved in hepatic cholesterol homeostasis* The expression of hepatic genes implicated in cholesterol synthesis, as well as export and degradation were investigated (Figure 7). A significant increase in sterol regulatory binding protein 2, *srebp2* (df = 2; F = 14.02; p < 0.01; Figure 7A), was observed in fish treated with 25 μg/g LNA-122i when compared to controls. No significant changes in the gene expression of hydroxymethylglutaryl CoA synthase, *hmgcs* (df = 2; F = 2.849; p > 0.05; Figure 7B), hydroxymethylglutaryl CoA reductase, *hmgcr* (df = 2; F = 0.864; p > 0.05; Figure 7C) and 7-dehydrocholesterol reductase*, dhcr7* (df = 2; F = 0.351; p > 0.05; Figure 7D), were observed. The expression of liver receptor x, *lxr (*df = 2; F = 4.5; p < 0.05: Figure 7E), increased in LNA-122i treated fish, irrespective of dose. No changes were observed in cholesterol-7alpha-hydroxylase a, *cyp7a,* (df = 2; F = 1.383; p > 0.05; Figure 7F), while *cyp7b* was increased in fish treated with 25 μg/g LNA-122i compared to other treatment groups (df = 2; F = 8.399; p < 0.01; Figure 7F). The expression of ATP-binding cassette sub-family G member 8, *abcg8* (df = 2; F = 1.175; p > 0.05; Figure 7G), did not change between treatment groups, while significant increases in the expression of ATP-binding cassette sub-family G member 5*, abcg5* (df = 2; F = 4.035; p < 0.05; Figure 7G) were found in trout injected with 25 μg/ g LNA-122i. The expression of UDP glycosyltransferase 1 family, polypeptide A3, *utg1a3* (df = 2; F = 6.79; p < 0.01; Figure 7H), increased in trout injected with either dose of LNA-122i.

#### Effect of omy-miRNA-122 inhibition on hepatic insulin signaling

**Figure 8:**
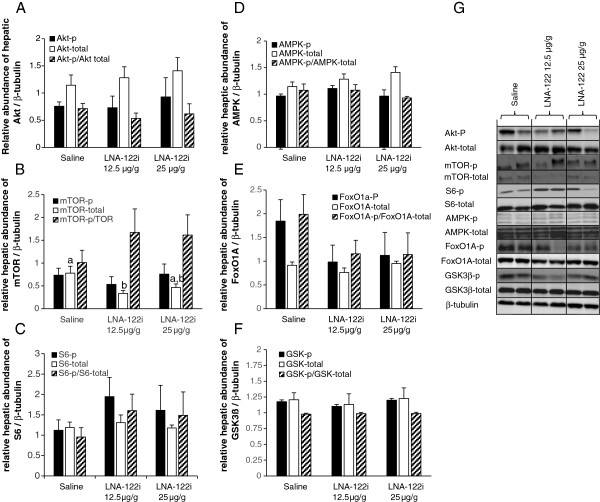
**Effect of LNA-122i treatment on hepatic abundance and phosphorylation status of key elements of the hepatic insulin signaling pathway.** Phosphorylated and total protein abundance was measured for AKT **(A)**, mTOR **(B)**, S6 **(C)**, AMPK **(D)**, FoxO1A **(E)**, GSK3β **(F)**. The individual graphs show the relative abundance of the phosphorylated form, the total protein and the ratio between the relative abundance of the phosphorylated form and the relative abundance of the total protein. All protein abundances were normalized by β-tubulin abundance, which did not change significantly with treatment (df = 2; F = 0.949; p > 0.05, data not shown).Representative Western Blot images are shown **(G)**. Analyzed groups consisted of n = 6 samples and data are depicted as mean ± S.E.M. for each group. Data were analyzed using one-way ANOVA and differences between individual experimental groups assessed by Student-Newman-Keuls post-hoc test. Different letters indicate a significant difference (p < 0.05) between experimental groups.

As an important upstream regulator of postprandial metabolic gene expression in the liver, we investigated the activity of the hepatic insulin signaling pathway. The activity of the insulin pathway, as determined by the ratio phosphorylated/total protein, remained unaltered for all components investigated (Figure 8A-G). Values from the statistical analysis are presented in Table 1. At the total protein level, a significant decrease in total mTOR protein was observed (df = 2; F = 5.285; p < 0.05; Figure 8B), which was significantly reduced in fish treated with 12.5 μg/g LNA-122i compared to saline-injected control fish.

**Table 1:** **Statistical analysis associated with the Western Blot measurements of (phosphorylated) proteins involved in the insulin signaling pathway and metabolic proteins shown in Figure **9**and**10

**Protein**	**One-way ANOVA**
AKT-P	df = 2; F = 0.130; p > 0.05
AKT-total	df = 2; F = 0.399; p > 0.05
AKT-P/AKT-total	df = 2; F = 0.461; p > 0.05
mTOR-P	df = 2; F = 0.444; p > 0.05
mTOR-total	df = 2; F = 5.285; p < 0.05*
mTOR-P/mTOR-total	df = 2; F = 0.445; p > 0.05
S6-P	df = 2; F = 0.786; p > 0.05
S6-total	df = 2; F = 0.278; p > 0.05
S6-P/S6-total	df = 2; F = 0.653; p > 0.05
AMPKα-P	df = 2; F = 1.070; p > 0.05
AMPKα-total	df = 2; F = 0.464; p > 0.05
AMPKα-P/AMPKα-total	df = 2; F = 0.643; p > 0.05
FoxO1-P	df = 2; F = 1.497; p > 0.05
FoxO1-total	df = 2; F = 0.974; p > 0.05
FoxO1-P/FoxO1-total	df = 2; F = 1.779; p > 0.05
GSK3β-P	df = 2; F = 0.145; p > 0.05
GSK3α + β total	df = 2; F = 0.339; p > 0.05
GSK3β-P/GSK3α + β total	df = 2; F = 0.145; p > 0.05
GK	df = 2; F = 1.652; p > 0.05
FAS	df = 2; F = 14.2; p < 0.001**
β-tubulin	df = 2; F = 0.409; p > 0.05

The asterisk symbol (*) indicates a significant difference at p < 0.05, while two asterisks (**) denote a significant difference at p < 0.001).

#### Effect of omy-miRNA-122 inhibition on hepatic protein abundance of key metabolic enzymes

**Figure 9:**
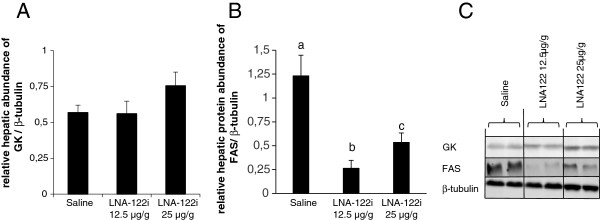
**Effect of LNA-122i treatment on hepatic protein abundance of key factors in hepatic glucose and lipid metabolism: Densitometry results for Glucokinase, GK (A) and fatty acid synthase, FAS (B) are shown, as well as representative Western Blot images (C).** Analyzed groups consisted of n = 6 samples and data are depicted as mean ± S.E.M. for each group. Data were analyzed using one-way ANOVA and differences between individual experimental groups assessed by Student-Newman-Keuls post-hoc test. Different letters indicate a significant difference (p < 0.05) between experimental groups.

Protein abundance of glucokinase, GK (Figure 9A), did not change significantly between treatment groups, while that of fatty acid synthase, FAS (Figure 9B), significantly decreased in fish injected with either dose of LNA-122i, compared to saline-injected control fish. Values from the statistical analysis are presented in Table 1.

## Discussion

The comparison of *miRNA-122* across several vertebrate species from genomic sequences reveals a complete conservation of *miRNA-122* and high conservation of miRNA-122* across vertebrate classes, while nucleotides forming the loop between both pairing strands in the *pre-miRNA-122* molecule appear to be under less selective pressure. The fact that *miRNA-122* is absent from the lamprey genome, but present in the elephant shark, an elasmobranch, places the acquisition of *miRNA-122* at the base of the vertebrate lineage evolution between 560 and 530 MYA [26]. Interestingly, and in contrast to other fish species, two loci for *pre-miRNA-122* containing two completely conserved mature *miRNA-122* sequences are present in the rainbow trout genome. This likely reflects tetraploidization and re-diploidization that occurred in the salmonid lineage 25–100 MYA [26], as two sequences were also identified in the genome of another salmonid species, the Atlantic salmon. However, the functional consequences of this duplication remain unknown, but provide for interesting future studies, especially given the relative rarity of miRNA duplication events in teleost fish genomes [27]. Being liver-specific and highly abundant in both rainbow trout [13,14] and mammals [4], the postulated hypothesis that *miRNA-122* evolved along with the vertebrate liver [16] is tempting. Interestingly, no *pre-miRNA-122* coding sequence was found in the lamprey genome. While a liver is already present in lampreys, which are believed to have separated from the vertebrate lineage 560 MYA [26], its phenotype is plastic across developmental stages, as it undergoes biliary atresia and lamprey develop a compensatory ability of the intestine to synthesize bile acids [28]. Since *miRNA-122* has been shown to play a role in hepatocyte and biliary tract differentiation in zebrafish [5,29], the evolution of *miRNA-122* may indeed have contributed to promote a stable hepatic phenotype seen in vertebrates. Our *in silico* target prediction revealed several specific trout mRNA targets of *miRNA-122*. Compared to a comprehensive list of predicted and validated targets from *miRNA-122* KO mice [30], a moderate 11% of predicted targets were conserved targets between both species. In spite of the moderate conservation of direct *miRNA-122* target genes between trout and mice, the identified conserved targets, in addition to newly identified trout-specific targets, are enriched for functional annotations indicative of cell proliferation and differentiation processes. Therefore, the functional prediction of *miRNA-122* target genes in rainbow trout is consistent with the development and maintenance of a hepatic phenotype, similar to the experimentally validated function of *miRNA-122* in mammals [6-8], and more recently, zebrafish [5,29]. This finding is further corroborated by the identification of rainbow trout mRNA targets of *miRNA-122* that are extra-hepatic and tissue specific, such as, for example, gastric chitinase, otholith matrix molecule 64, muscle-expressed myosin light chain, blood cell-expressed hemoglobin, and retina-expressed rhodopsin (Additional file 1). This is in good agreement with the postulated role of the tissue-specific *miRNA-122* which may in part maintain the hepatic phenotype by repressing extra-hepatic tissue-specific transcripts [31]. With the exception of glucose metabolism (Additional file 1), little enrichment was observed for predicted rainbow trout *miRNA-122* target genes with a role in metabolic pathways. This is consistent with the finding that metabolic effects of *miRNA-122* may be related to ‘indirect’ *miRNA-122* target genes, whose expression is modulated indirectly and as a consequence of alteration of gene expression of direct target genes. For example, several *miRNA-122* inhibition studies in mammalian model systems have linked the observed decrease in cholesterol and triglycerides in these species to decreased expression of cholesterol and fatty acid synthesis genes [9-11], without establishing a link to direct *miRNA-122* target genes. Therefore, in addition to validating individual predicted direct *miRNA-122* target gene expression to assess functional efficiency of *miRNA-122* inhibition in trout, we focused on analyzing the expression of non-target markers of key hepatic metabolic pathways to elucidate the underlying (indirect) molecular mechanisms for the observed metabolic consequences *in vivo*. This approach is especially suitable in studying metabolic pathways, which, albeit not exclusively, are highly regulated at the level of transcription [32]. It should be noted, however, that 3′UTR annotation of trout mRNAs remains incomplete, and that future studies may identify additional (metabolic) *miRNA-122* target genes in rainbow trout.

### Efficient miRNA-122 isomiRNA inhibition in rainbow trout in vivo

Inhibition of *miRNA-122* was confirmed by *real-time* RT-PCR, which has been shown to be a monitoring technique to assess efficient hepatic *miRNA-122* inhibition *in vivo*[10,11,33]. Given the fact that multiple isomiRNAs of *miRNA-122*, likely resulting from posttranscriptional modifications, had initially been described in both rainbow trout as in mammals [13,34], we aimed to inhibit the expression of not only the conserved mature *omy-miRNA-122*, but also of the identified trout isomiRNAs, specifically *omy-miRNA-122a* and *omy-miRNA-122b*. The *miRNA-122* isomiRNAs share a common *miRNA-122* seed sequence, hence all are considered functional. However, possible divergent biological functions for the isomiRNAs are currently debated [34,35]. With regard to the study of regulatory effects of *miRNA-122* in rainbow trout, the fact that all three isoforms were found to be inhibited by LNA-122i treatment ensures that no uninhibited functional isoforms of *miRNA-122* can compensate for the inhibited mature form. In order to exclude potential effects of the LNA-122i treatment on the canonical miRNA pathway, we assessed the expression of non-targeted *omy-miRNA-103*, *omy-miRNA-21* and *omy-miRNA-33*, and found no significant changes in expression between treatment groups. Lastly, as an index of functional inhibition of *miRNA-122*, we validated an expected de-repression of *in silico* predicted, rainbow trout-specific targets of *miRNA-122*, including the fish specific cytochrome *cyp2k5*, the prostaglandin reductase *ptgr1*, and archain *arcn1*. For all three genes, a significant de-repression was confirmed, irrespective of the dose administered. Overall, our study reveals the feasibility of silencing approaches in fish, an area comparatively understudied [36,37], in spite of its great potential for comparative physiology.

### Inhibition of miRNA-122 results in postprandial hyperglycemia and decreased availability of lipids in the plasma

The dose-independent >60% increase in blood glucose found in trout subjected to *miRNA-122* inhibition has not been described in mice, where *miRNA-122* inhibition resulted only in slight, but non-significant, trends for increases in plasma glucose [9]. Interestingly, in an acute glucose tolerance test in *miRNA-122* knock-out mice, Tsai and colleagues [30] observed small, but statistically significant increases in acute postprandial (60–120 min) measurements of blood glucose after glucose injection, emphasizing the importance of sampling time when analyzing particular metabolic endpoints. Nevertheless the increase appears to be stronger in rainbow trout than in mammalian model systems, especially given the fact that *miRNA-122* knock-out mice generally suffer from more pronounced metabolic consequences compared to mice injected with a LNA-122i which display reversible metabolic effects [11,30]. With regard to plasma triglyceride concentration, the significant >30% decrease in plasma triglyceride concentration in trout treated with 25 μg/g LNA-122i is similar to a significant 40% decrease observed in mice treated with the same dose of LNA-122i and subjected to a similar injection protocol [10]. Similarly, recent studies on *miRNA-122* knock-out models detected significant, persistent decreases in serum triglycerides [30]. The concentration of free fatty acids in trout plasma paralleled the observed plasma concentration of triglycerides. The plasma concentration of free fatty acids decreased significantly (<40%) in trout injected with the higher dose of 25 μg/g of LNA-122i. This is in contrast to findings in mammalian model systems, where only a very slight, non-significant decrease of free fatty acids in mice injected with *miRNA-122* inhibitors have been found [9]. A decrease of 20% in plasma cholesterol concentration was observed in trout injected with LNA-122i, irrespective of the dose administered. This decrease in plasma cholesterol concentrations was significant but slight, and post-hoc comparisons did not resolve differences between individually compared treatment groups. In mammalian *miRNA-122* inhibition studies [9-11], as well as recent mammalian knock-out models [30,38], reductions of cholesterol between 20-50% have been observed, making it the most consistently observed metabolic effect of *miRNA-122* inhibition in mammals. In all cases, the effects on plasma cholesterol are long lasting but reversible [11] and interestingly, similar to our study, an increase in dose does not lead to further decrease in plasma cholesterol concentration, suggesting a saturation effect [10]. While the metabolic effects of *miRNA-122* inhibition in trout are largely in line with the effects reported in the mammalian literature, the quantitative nature of these effects appears to differ slightly between trout and mammalian model systems. Nevertheless, these differences should be interpreted with caution, since the measured metabolites are subject to acute, but temporally different, postprandial changes in mammals [39] as in trout [40]. Hence, the fact that our study was specifically designed to target acute postprandial changes is in contrast to most published literature using mammalian model systems, where animals were either fasted, or the information of the feeding times was not indicated [9-11]. These differences in experimental design may have contributed to different measurements of plasma metabolite concentrations.

### Characterization of potential hepatic molecular mechanisms underlying the metabolic phenotype

In order to establish potential underlying molecular mechanisms in the development of the observed metabolic phenotype in rainbow trout experiencing *miRNA-122* inhibition, we investigated specific metabolic markers in the hepatic tissue.

### Postprandial hyperglycemia in trout with miRNA-122 inhibition decreases hepatic gk expression and FAS protein levels

To investigate molecular mechanisms underlying the observed postprandial hyperglycemia, we investigated hepatic gene expression for transcripts implicated in the hepatic intermediary metabolism of glucose and lipids, as transcriptional regulation of these pathways plays an important role in regulating systemic glucose homeostasis [32]. Specifically, we investigated hepatic genes with a role in (1) glucose utilization (glucose uptake, glycolysis and glycogenesis) and (2) hepatic glucose production (gluconeogenesis and glycogenolysis). Additionally, to test our hypothesis that *miRNA-122* alters glucose metabolism by regulating hepatic *de novo* lipogenesis, we quantified responses for genes involved in lipogenesis and fatty acid oxidation. The effect of *miRNA-122* inhibition on hepatic protein abundance of key enzymes of both, the glycolytic (GK), and lipogenic pathway (FAS) were measured, to account for potential effects of *miRNA-122* at the protein level in these pathways.

With respect to hepatic glucose catabolism, we identified a significant decrease in *gk* mRNA expression in fish when injected with 25 μg/g LNA-122i. Glucokinase belongs to the hexokinase family and is highly expressed in the liver; its specific properties allow the hepatic influx of glucose across the physiological range of blood glucose concentration [41]. Functionally, glucokinase therefore represents an important node in glucose metabolism, channeling postprandial hepatic glucose flux towards oxidative pathways, but also towards energy storage in the form of glycogen deposition and *de novo* lipid synthesis, the latter of which is subsequently exported to adipose tissue for storage. It can therefore be considered the first step of lipogenesis. Liver specific knock-out [42] or overexpression [43,44] of *gk* in mammalian model systems provide unequivocal evidence that hepatic GK regulates blood glucose homeostasis by limiting hepatic glucose utilization for glycogen synthesis and the *de novo* lipogenic pathway. In rainbow trout, *gk* expression is, as in mammals, mainly hepatic, and is reduced by fasting and induced by (carbohydrate) feeding [45]. Interestingly, in trout, unlike in mammals, carbohydrates are capable of stimulating *gk* expression independently of insulin [46,47], and thus *gk* is considered a glucose sensor [46,47]. The diminished levels of *gk* mRNA may be indicative of a reduced postprandial glucose sensing capacity in trout experiencing *miRNA-122* inhibition. However, the extrapolation of functional consequences, as seen in mammalian knock-out models is difficult based on our data, since, at the protein level, hepatic GK abundance did not change between control and LNA-122i injected fish. Further time course studies are required to address whether the decreased postprandial induction of the glucosensor *gk* results in temporally delayed changes in GK protein concentration. With regard to glycogen synthesis, a paradoxical increase in mRNA abundance of hepatic glycogen synthase, the rate-limiting enzyme in glycogen deposition, was observed in trout injected with either dose of LNA-122i. While these data do not correspond to the observed increase in plasma glucose, the fact that fish were sampled 5 d following the last injection of the LNA-122i may reflect that the induction of *gys2* represents a counter-regulatory response to cope with hyperglycemia. The observed change in indicators of glycogen metabolism, particularly in the form of increased *gys2* expression, is in contrast to findings from a recent mouse *miRNA-122* knock-out model, where mild postprandial hyperglycemia was correlated with decreased hepatic glycogen storage and decreased protein abundance and activity of glycogen synthase [30]. Unfortunately, due to the limited hepatic size in juvenile fish, we were unable to use the samples to measure hepatic glycogen content directly in addition to other hepatic measurements, therefore the current results should be interpreted with caution. Transcript markers of hepatic anabolic pathways of glucose metabolism, specifically at the level of gluconeogenesis and glycogenolysis, did not change with LNA-122i treatment, indicating that the observed postprandial hyperglycemia in LNA-122i treated rainbow trout is, at the level of gene expression, not related to hepatic glucose production or liberation.

In terms of lipid metabolism, *miRNA-122* inhibition in trout resulted in significant increases in the expression of genes involved in lipogenesis (*srebp1c*, *acc*). The expression pattern observed for lipogenic genes is in contrast to mammalian studies, where comparable *miRNA-122* inhibition results in decreased expression of lipogenic genes, which correlate with decreased lipogenesis. Similarly, the observed results are contrary to correlative evidence from postprandial studies in rainbow trout [14], in which a positive correlation between *omy-miRNA-122b* and the lipogenic genes *srebp1c* has been described. Whether these differences represent distinct, species-specific actions of *miRNA-122* in trout and mammalian models in the form of a direct regulation of *srebp1c* or *acc* by miRNA-122 in trout can currently not be predicted, as 3′UTR sequences for either gene are currently unavailable. Differences in the experimental protocol may also play a role in the observed changes at the level of gene expression, especially given that the metabolic consequences on plasma lipid metabolites are largely consistent between trout and mammals. While most studies in mammalian models investigated gene expression shortly after the last injection [9,10], our study design measured effects several days following the last injection. In line with this, the only transcriptomic time-course study of *miRNA-122* inhibition in the liver of mice [11], revealed an initial inhibition of the expression of the lipogenic gene *srebp1c*, which however were not detected 1 wk following the treatment, in spite of persisting, albeit less severe, *miRNA-122* inhibition and metabolic effects. The increased expression of *acc,* the rate limiting enzyme in lipogenesis with both doses of LNA-122i, may represent an adaptive response to cope with increased glycemic load, similar to the effect observed for *gys2*. Whether these mechanisms do indeed represent physiological responses to maintain homeostasis in plasma metabolites is not known, but may be delineated by following a time-course study, and through aforementioned advances in annotation of 3′UTR sequences in trout. While we did not observe changes in *fas* gene expression between treatment groups*,* hepatic FAS abundance was significantly inhibited at the protein level, similar to *miRNA-122* KO mice [30]. While this is indicative of reduced hepatic lipogenesis that is also observed in mammalian model species [10], the current result does not support the previously observed post-prandial and insulin-mediated co-regulation of miRNA-122 and *fas*[15]. The discrepancy between gene expression and protein abundance data suggests that, in spite of the previously observed co-regulation of specific *miRNA-122 isomiRNAs* and *fas* in trout [14,15] and the observed concurrent decrease of *fas* in miRNA-122 inhibited mammalian models [11], *fas,* at the level of gene expression, may not be an indirect target of miRNA-122 in rainbow trout. Alternatively, the LNA-122i induced decrease of FAS, but not *fas* gene expression, may reflect temporally anterior changes in *fas* expression/translation in unfed fish, which based on the estimated half-life of FAS in mammals [48], may still manifest themselves postprandially. As previously noted, the limited amount of biological material in juvenile fish prevented the direct measurement of hepatic lipid contents, and without direct measurements of hepatic lipid concentrations in addition to the measured plasma lipid concentrations, the current interpretations on hepatic lipogenesis are inferred from hepatic gene expression and protein data, as well as plasma metabolite data. Therefore these data should be interpreted cautiously.

### Inhibition of omy-miRNA-122 does not alter hepatic insulin signaling

Since *miRNA-122* has been shown to stimulate hepatic insulin signaling in mammals [49], and since a postprandial coordination of glycolysis and lipogenesis is mediated by the insulin pathway in rainbow trout [50], as in mammals [51], we investigated the possible upstream involvement of the insulin pathway in the observed metabolic effects of *miRNA-122* inhibition in rainbow trout. In trout, similar to the situation in mammals, it has recently been shown that inhibition of mTOR, a key node in the insulin pathway, results in decreased expression of hepatic *gk* and *fas*[50]. Given our hypothesis that *miRNA-122* may control glucose homeostasis through regulation of glycolytic flux and subsequent *de novo* lipogenesis in rainbow trout, we analyzed the postprandial activity of hepatic insulin pathway with a particular focus on the mTOR node. Indeed, recent evidence from studies investigating *miRNA-122* function in mammalian model systems points to a stimulatory role for *miRNA-122* on the activity of the insulin pathway, and mTOR in particular. Depletion of *miRNA-122* in Hep2 cells resulted in tyrosine phosphatase 1B induction and subsequently, reduced activity of the insulin pathway, including a reduction in mTOR phosphorylation status [49]. Inhibition of *miRNA-122* equally resulted in increased phosphorylation status of the metabolic sensor AMPKα [10], which, in its phosphorylated form, acts to inhibit mTOR signaling [52]. In our study, *miRNA-122* inhibition resulted in no notable differences in the phosphorylation status of any component of the hepatic insulin signaling pathway, indicating that the metabolic effects observed in trout injected with LNA-122i are not mediated by acute, postprandial alteration of hepatic insulin signaling. Interestingly, the only detected change in any of the components of the insulin pathway was noted in the total protein abundance of mTOR, which decreased significantly in trout injected with 12.5 μg/g LNA-122i. This regulation however did not alter the activity of the mTOR kinase, since the ratio of mTOR phosphorylated form/total form did not change significantly between treatment groups and since the same index was unaffected for S6, a downstream target of mTOR. While the physiological relevance of a decrease in total hepatic mTOR protein abundance is difficult to interpret, it may limit the maximum capacity of downstream events such as lipogenesis. Whether such decreases in total mTOR abundance can become rate-limiting under physiological conditions, however, is currently not known.

### Genes involved in reverse cholesterol transport are increased in miRNA-122-inhibited fish

The inhibition of *miRNA-122* resulted in slightly, but significantly, reduced plasma cholesterol levels, mirroring findings from numerous studies in mammalian models [9-11,30]. In contrast to the identification of a decrease in cholesterol biosynthesis genes in these studies, which were considered to be a likely cause for the observed decrease in plasma cholesterol, genes implicated in cholesterol synthesis either increased (*srebp2*) or did not change their expression (*dhr7*; *hcgr*; *hcgs*) in response to *miRNA-122* inhibition in trout. Conversely, *miRNA-122* inhibition resulted in an increase in genes implicated in cholesterol sensing (*lxr*), and excretion (*abca5*, *utg1a3*). Interestingly, the expression of *lxr*, a nuclear receptor that serves as a rheostat for systemic cholesterol homeostasis [53-55], was increased in LNA-122i injected trout, irrespective of dose. In the liver, LXR is activated by its endogenous ligand oxysterol, which is generated in conditions of high intracellular concentrations of cholesterol [56]. In a positive feedback loop, activated LXR stimulates its own expression, which is the case in both mammals [57] and rainbow trout [58]. In mammals, hepatic LXR regulates several effectors involved in excreting cholesterol directly into the bile, or metabolizing cholesterol into bile acids, which are subsequently conjugated and excreted via bile secretion [54]. Given the conservation of several targets of LXR between mammals and fish [59,60], we investigated the potential induction of these downstream targets to provide further evidence for an activation of LXR, and indeed detected an increase in *utg1a3,* a bile conjugating enzyme with a proposed role in cholesterol elimination [61,62]. Interestingly, a tendency for facilitated biliary clearance has been measured in *miRNA-122* knock-out mice, however there was a low treatment number in the study and a statistical difference was not detected [30]. The observed increase in *lxr* and putative activity of LXR may be related to the observed hyperglycemia, since some studies implicate LXR as a glucose sensor, which binds glucose with similar affinity as oxysterols [63]. Similar to the previously discussed metabolic markers, time course studies are needed to delineate potential time-specific effects from true species-specific differences for the regulation of genes implicated in cholesterol metabolism. For example, while the expression of several genes implicated in cholesterol biosynthesis was decreased 1 d following the last injection of LNA-122i in mice, this effect was not observed 1 wk following the last injection of LNA-122i, in spite of a stably maintained decrease in plasma cholesterol concentrations [11].

## Conclusion and future perspectives

Inhibition of *miRNA-122* in rainbow trout results in metabolic changes that are qualitatively similar to changes observed in mammalian models. However, quantitative differences, for example in postprandial glucose concentrations, may represent species-specific differences, which appear to be more pronounced in trout compared to previous mammalian studies. Mechanistically, the increased hyperglycemia does not appear to be related to hepatic glucose supply, favoring the hypothesis of decreased hepatic glucose utilization. Indeed, the signature of specific components, notably reductions in liver *gk* expression, as well as reduction of hepatic FAS protein abundance, are in line with the proposed hypothesis that *miRNA-122* regulates glucose homeostasis via modulation of glycolytic flux towards *de novo* lipogenesis. The regulation of these genes appears to be independent of the insulin signaling pathway, and is likely related to as of yet unidentified direct targets. Our *in silico* analysis of predicted *miRNA-122* targets in trout revealed a strong enrichment for cell cycle, proliferation and differentiation processes. Given that these *miRNA-122* targets are conserved between trout and mice, and that cell-cycle regulators are proposed to cross-talk with metabolic pathways [64], genes involved in this group may be good candidates for mediating metabolic effects. As well, predicted *miRNA-122* targets in trout were enriched for functions in glucose metabolism (insulin growth factor 2, *igf2*; atrial natiuretic peptide, *anp*; interleukin-15 receptor alpha chain, *il15ra*; uncoupling protein 2, *ucp2*; glucose transporter 1a, *glut1a*; growth hormone 2, *gh2*; cellular differentiation marker 80, *cd80;* suppressor of cytokine signaling 6*, socs6*), a result that may indicate direct regulation of glucose metabolism by *miRNA-122* in trout. However, the distinct roles of these genes in trout have not been characterized with regard to glucose metabolism, but present an interesting avenue for future study. While our study is the first to characterize metabolic effects of the conserved *miRNA-122* in a non-mammalian vertebrate, future detailed time-course studies are needed to fully differentiate between true species-specific differences, and time-dependent effects of *miRNA-122* action, especially given that the metabolic functions of *miRNA-122* have been shown to underlie circadian regulation in mammals [65].

## Methods

### In silico approaches

#### Analysis of pre-miR-122 sequences in fish and vertebrate genomes

Sequences of *miRNA-122* were obtained by BLAST analysis of the zebrafish *pre-miRNA-122* genome sequence against genome sequences available in the ENSEMBL database (http://www.ensembl.org). Following this approach we retrieved *pre-miRNA-122* sequences from the African clawed frog (*Xenopus leavis)* [ENSXETG00000028985], the Carolina anole (*Anolis carolinensis)* [ENSACAT00000018998], the red jungle fowl (*Gallus gallus* [ENSGALG00000018343], the human (*Homo sapiens)* [ENSG00000207778], the field mouse (*Mus musculus)* [ENSMUSG00000065402], the West Indian ocean coelacanth (*Latimeria chalumnae)* [JH127895.1 9514 to 9584 (+)]*,* the Atlantic cod (*Gadhus morua*) [contig56666 645 to 717 (+)], the green spotted puffer (*Tetraodon nigroviridis*) [ENSTNIT00000023441], the fugu (*Takifugu rupipres*) [ENSTRUG00000018785], the three-spined stickleback (*Gasterosteus aculeatus*) [group XIV 3950425 to 3950502 (+)], the Japanese rice fish (*Oryzias latipes)* [ENSORLG00000020901] and the common platy (*Xiphophorus maculatus)* [JH556689.1 363573 to 363653 (+)]*.* The same approach was taken for the elephant shark (*Callorhinchus milii*) [AAVX01098255.1] using the elephant shark genome project (http://catfishgenome.imcb.astar.edu.sg), the catfish (*Ictalurus punctatus*) [FI859091], using the catfish genome database (http://www.catfishgenome.org/catfish/cbarbel), the Atlantic salmon (*Salmo salar*), using the salmon database (http://genomicasalmones.dim.uchile.cl) [AGKD01016205.1 and AGKD01081167.1], the rainbow trout, using the INRA rainbow trout genome resources (Y. Guiguen, personal communication), and the gilthead sea bream (*Sparus aurata*) [AM950993.p.sb.5], using the INRA Sigenae database (http://www.sigenae.org), respectively. The genomic *pre-miRNA-122* sequences were aligned using ClustalW2 (http://www.ebi.ac.uk/Tools/msa/clustalw2).

### Prediction of omy-miR-122 mRNA targets

Compared to species whose genomes have been completely assembled and annotated, predictions of miR-122 binding sites in 3′UTRs of rainbow trout suffer from the caveat that only a limited number of 3′UTRs have been published in rainbow trout. This problem is exacerbated by the presence of teleost genome duplications, resulting in a higher number of protein-coding genes compared to higher vertebrates [66]. Nevertheless, since miRNA-target relationships may undergo substantial species-specific evolutionary changes [67], we obtained available annotated rainbow trout 3′UTR sequences (n = 1059) from the UTR database (http://www.utrdb.ba.itb.cnr.it)*,* in order to assure a species-specific target prediction*.* Of the retrieved annotated rainbow trout *3′*UTRs, we selected sequences that contained a perfect seed match (n = 83) corresponding to nucleotides 2–7 of the *miRNA-122*. This approach was chosen, since the sequence of *miRNA-122* is completely conserved in vertebrate evolution (Figure 1), implicating the same seed is functional in trout as in mammals. In studies using mammalian models, a 2–3 fold enrichment for this sequence motif has been shown in the 3′UTRs of up-regulated mRNA following *miRNA-122* inhibition [9,11], and, similarly, in the 3′UTR of mRNA transcripts corresponding to identified upregulated proteins following *miRNA-122* inhibition [31]. To gain insight into the potential functional roles of these predicted targets in rainbow trout, we identified human homologous sequences using Uniprot ID (http://www.uniprot.org/). This approach resulted in the successful mapping of 76 predicted rainbow trout target genes to mammalian homologs. Based on these identified mammalian homologs, a sub-network enrichment analysis (SNEA) was performed in Pathway Studio 9.0 (Ariadne, Rockville, MD, USA) and ResNet 9.0. SNEA was performed to identify gene networks that were significantly enriched with whose mRNAs contained predicted *miRNA-122* target sites. Briefly, SNEA builds sub-networks starting from a central seed from molecular relationships (e. g., expression or binding). These data are retrieved from the ResNet 9 database, which is compiled by Ariadne using the MedScan database. The MedScan database contains over 20 million PubMed abstracts and approximately 900 K full-text articles. This is followed by a statistical comparison between the sub-network and a background distribution of known gene networks using a Mann–Whitney U-Test, generating a p-value that indicates the statistical significance of the difference between these two distributions (additional details on the method can be found in the technical bulletin p. 717 from Pathway Studios 7.0). The enrichment *p*-value was set at *p* < 0.05. This approach has been previously applied for the identification of gene and protein networks in teleost fishes [68,69].

### Fish and experimental design

**Figure 10:**
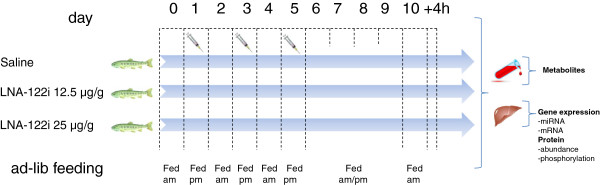
**Schematic representation of the experimental design for the *in vivo *study of metabolic effects of LNA-122i.** Detailed explanations are provided in the text. Samples analyzed in this study stem from postprandial fish fed 4 h before sacrifice.

The experiments were carried out in accordance with the clear boundaries of EU legal frameworks, specifically those relating to the protection of animals used for scientific purposes (i.e. Directive 2010/63/EU), and under the French legislation governing the ethical treatment of animals (Decret no. 2001–464, May 29th, 2001). The investigators carrying out the experiment had “level 1” or “level 2” certification, bestowed by the Direction Départementale des Services Vétérinaires (French vetinary services) to carry out animal experiments (INRA 2002–36, April 14th, 2002). The experiment was conducted at INRA facilities in Donzaq, certified for animal services under the permit number A64.495.1 by the French vetinary services, which is the competent authority. Trout eggs were incubated until hatching and alevins reared in 8˚C stream water at the INRA experimental facility in Les Athas, France. Once fish reached the juvenile stage, animals were transferred to the INRA experimental facility at Donzacq, France, where they were maintained in 18˚C oxygenated spring water. Juvenile trout (n = 6) with an average weight of 20 g were distributed in three 50 l tanks and subjected to 3 intraperitoneal (i.p.) injections per week of either teleost physiological saline (0.6%), or 12.5 μg/g and 25 μg/g body weight of LNA-122i (Locked Nucleic Acid *miRNA-122* inhibitor; Exiqon, Vedbæk, Denmark) respectively, dissolved in physiological saline (Figure 10). The LNA-122i is an oligonucleotide with the specific sequence 5′ ATTGTCACACTCC 3′ which contains phosphorothioate backbones to improve *in vivo* stability and distribution [70]. A short (13 nt) LNA-122i was chosen to inhibit all described rainbow trout *miRNA-122* isomiRNAs. In addition to the completely conserved *omy-miRNA-122* identified from trout genomic sequences (Figure 1), additional isomiRNAs sharing the same functional seed sequence, but differing in their 3′nucleotides have been identified through next generation sequencing in trout, likely resulting from posttranscriptional modification of the primary transcript [13]. Therefore, in an effort to inhibit all *omy-miRNA-122* isomiRNAs, an inhibitor was chosen which contains the reverse complement of the seed region (nucleotides 2–7 of all identified *omy-miRNA-122* isomiRNAs, but does not discriminate between alternate 3′ nucleotide sequences. Trout were not fed prior to injections and, following the last injection, rainbow trout were fed ad libitum in the morning and evening for 4 days. On the fifth day, fish were fed in the morning and sacrificed four hours after the meal, and liver tissue was collected and flash frozen in liquid nitrogen and subsequently stored at −80˚C until further analysis. This postprandial timeframe for the experimental protocol was based on previous studies in rainbow trout, that had revealed a postprandial increase in hepatic expression of *omy-miRNA-122b* isomiRNA in rainbow trout 4 h after feeding [14], in addition to activation of the hepatic insulin pathway and regulation of metabolic gene expression. The number and doses of the LNA-122i injections were based on previous studies in mice [11], which revealed functional inhibition of *miRNA-122* and associated metabolic effects at doses between 12.5 μg/g and 25 μg/g up to one week after the last injection.

### Plasma metabolic profile

Plasma glucose (Glucose RTU, BioMérieux, Marcy l′Etoile, France), triglycerides (PAP 150, BioMérieux), free fatty acids (NEFA C kit, Wako Chemicals, Neuss, Germany), and cholesterol (Cholesterol RTU, BioMérieux) concentrations were determined using commercial kits adapted to a microplate format, according to the recommendations of the manufacturer.

### Total RNA extraction and cDNA synthesis

Relative hepatic gene expression was determined by quantitative real-time RT-PCR. The extraction of total RNA was performed using the Trizol reagent (Invitrogen, Carlsbad, CA, USA) according to the manufacturer’s instructions. An amount of 1 μg of total RNA was used for cDNA synthesis. The NCode™ VILO™ miRNA cDNA synthesis kit (Invitrogen), or the SuperScript III RNAse H– Reverse transcriptase kit (Invitrogen) with random primers (Promega, Charbonniéres, France), were used to synthesize cDNA (n = 6 for each treatment) for miRNA and mRNA, respectively.

### Real-time RT PCR

For gene expression assays, forward primer sequences for miRNAs were taken directly from the sequence information provided by Salem and colleagues [13], or, if not available, designed based on miRNA sequences found in the trout genome. The universal reverse primer used for all miRNA expression analysis was provided by the manufacturer with the NCode™ VILO™ miRNA cDNA synthesis kit (Invitrogen). The primer sequences used in the real-time RT-PCR assays for miRNAs and metabolic genes, as well as the conditions of the assays have been previously described [14,40,58,71]. For gene targets that had not been previously validated, primer sequences, specific assay conditions and available accession numbers from Genebank (http://www.ncbi.nlm.nih.gov/genbank) or the INRA trout EST database SIGENAE (http://www.sigenae.org) are shown in Table 2. For real-time RT-PCR assays of miRNAs, the Roche Lightcycler 480 system was used (Roche Diagnostics, Neuilly-sur-Seine, France). The assays were performed using a reaction mix of 6 μl per sample, consisting of 2 μl of diluted cDNA template, 0.12 μl of each primer (10 μM), 3 μl Light Cycler 480 SYBR® Green I Master mix, and 0.76 μl DNAse/RNAse free water (5 Prime GmbH, Hamburg, Germany). The PCR protocol was initiated at 95°C for 10 min for initial denaturation of the cDNA and hot-start Taq-polymerase activation, followed by 45 cycles of a two-step amplification programme (15s at 95°C; 40 s at 60–64°C), according to the primer set used. Melting curves were systematically monitored (temperature gradient at 1.1°C/10 s from 65–94°C) at the end of the last amplification cycle to confirm the specificity of the amplification reaction. Each PCR assay included replicate samples (duplicate of reverse transcription and PCR amplification, respectively) and negative controls (reverse transcriptase- and cDNA template-free samples). The gene expression assays used for protein-coding genes have been described previously [14]. As for omy-miRNA SYBR Green assays, melting curves were systematically monitored (temperature gradient at 0.5°C/10 s from 55–94°C at the end of the last amplification cycle), to confirm the specificity of the amplification reaction. Each PCR run included replicate samples and controls as described above. The specificity of reactions used to amplify previously uncharacterized amplicons was further confirmed by sequencing of the PCR product (Beckman-Coulter, Hope End Takeley, Essex, UK), followed by a BLAST search of the obtained sequences (http://blast.ncbi.nlm.nih.gov/Blast.cgi). For the expression analysis of miRNA and mRNA, relative quantification of target gene expression was performed using gene expression values of *u6* and *ef1α* for the normalization of measured hepatic miRNAs and mRNAs, respectively, as neither gene expression values changed significantly between treatment groups (data not shown). In all cases, PCR efficiency (E) was measured by the slope of a standard curve using serial dilutions of cDNA and PCR efficiency values ranged between 1.8 and 2.2, corresponding to 90-110% efficiency.

**Table 2:** Sequences and conditions for new primers used in SYBR Green real-time RT-PCR assays

**Gene**	**Forward primer (5′3′)**	**Reverse primer (3′5′)**	**Tm (°C)**	**Gene bank or SIGENAE accession number**	**Amplicon size (bp)**
*omy-miRNA-122*	TGGAGTGTGACAATGGTGTTTGT	Poly T Primer (Invitrogen)	60	-	-
*cyp2k5*	CTCACACCACCAGCCAAGAC	CGTCAGCAGAGGTAACACATCAG	60	NM_001124742	76
*ptgr1*	CAGTGATTGTGGATGGAGGAG	CCTTGATCTTGGCGATCTGT	60	NM_001124634	238
*arcn1*	AGCCTCACTTGTGGGAGAGA	CTTGTCACCGTTGTTGATGG	60	NM_001164069	119
*Gs*	AAGATATGGAGGCGGAGAGG	GATCTCAAGGACCAGGGTTG	60	BX299627.p.om.8	152
*Pygl*	AACCGACACCTCCACTTCACC	CCTGCATCTTCCTCCATCTC	60	BX882218.p.om.8	296
*srebp2*	TAGGCCCCAAAGGGATAAG	TCAGACACGACGAGCACAA	60	BX859204.p.om.8	161
*Hmgcs*	AGTGGCAAAGAGAGGGTGTG	TTCTGGTTGGAGACGAGGAG	60	CX251971.p.om.8	298
*Hmgcr*	GAACGGTGAATGTGCTGTGT	GACCATTTGGGAGCTTGTGT	60	AB218825.p.om.8	216
*dhcr7*	GTAACCCACCAGACCCAAGA	CCTCTCCTATGCAGCCAAAC	60	CA376644.p.om.8	289
*cyp7a*	ACGTCCGAGTGGCTAAAGAG	GGTCAAAGTGGAGCATCTGG	60	AB675933	111
*cyp7b*	ACAGAGACCTCACCTTCACCA	GATCTCCCTTCCTCACTCCA	60	F6VG15V01BFI5C.p.om.8	171
*abcg5*	CACCGACATGGAGACAGAAA	GACAGATGGAAGGGGATGAA	60	CU073172.p.om.8	268
*abcg8*	GATACCAGGGTTCCAGAGCA	CCAGAAACAGAGGGACCAGA	60	FYV3OTN01BVHON.p.om.8	159
*utg1a3*	CCACCAGCAAGACAGTCTCA	CAACAGCACAGTGGCTGACT	60	F6VG15V01BF6CZ.p.om.8	216

Gene bank accession number (http://www.ncbi.nlm.nih.gov/genbank) are provided when available. For the remaining sequences, accession numbers from the trout EST database SIGENAE (http://www.sigenae.org) are provided. Additional primer sequences have been previously published and can be found in sources cited in the text.

### Western blotting

Frozen liver samples (~300 mg) were homogenized on ice with an Ultraturrax homogenizer (IMLAB Sarl, Lille, France). During homogenization, samples were kept in a buffer containing 150 mmol l^−1^ NaCl, 10 mmol l^−1^ Tris, 1 mmol l^−1^ EGTA, 1 mmol l^−1^ EDTA (pH 7.4), 100 mmol l^−1^ sodium fluoride, 4 mmol l^−1^ sodium pyrophosphate, 2 mmol l^−1^ sodium orthovanadate, 1% (v/v) Triton X-100, 0.5% (v/v) NP40-IGEPAL, and a protease inhibitor cocktail (Roche, Basel, Switzerland). Homogenates were centrifuged at 1000 g for 30 min at 4°C, and supernatants were then centrifuged for 45 min at 15.000 g. The resulting supernatants (n = 6 for each time point) were stored at −80°C. Protein concentrations were determined using the Bio-Rad Protein assay kit (BIO-RAD, Hercules, CA, USA). According to the protein, quantities of 5–20 μg protein per sample were subjected to SDS-PAGE and Western Blotting, using the appropriate antibodies. All primary antibodies used for analysis of the insulin signaling pathway were obtained from Cell Signaling technologies (Ozyme, Saint Quentin Yvelines, France), while antibodies used for the measurement of GK, and FAS were obtained from Santa Cruz Biotechnology (Santa Cruz, CA, USA). All antibodies have been shown to cross-react successfully with rainbow trout proteins of interest [21,40,50,72,73]. All primary antibodies used were raised in rabbit, and after final washing, membranes were incubated with an IRDye infrared secondary anti-rabbit antibody raised in goat (LI-COR Inc. Biotechnology, Lincoln, NE, USA). Bands were visualized and quantified by Infrared fluorescence using the Odyssey® Imaging System (LI-COR Inc. Biotechnology, Lincoln, NE, USA).

### Statistical analysis

Data were analyzed by univariate ANOVA. In cases where data were nonparametric or not homoscedastic, data transformations were used to meet ANOVA criteria. Normality was assessed using the Shaprio-Wilk test, while homoscedasticity was determined using Levene’s test. Following univariate ANOVA analysis, The Student-Newman-Keuls test was used for post-hoc analysis. Data were analysed using the R software/R Commander package.

## Competing interests

The authors declare that they have no competing interests.

## Authors’ contributions

JAM, SP, IS, SS designed the study. JAM retrieved genomic pre-mir-122 sequence data and rainbow trout UTR data. JAM performed pre-miRNA-122 sequence alignments and CJM performed the *in silico* analysis of predicted miRNA-122 trout target genes. JAM performed the metabolite, real-time RT-PCR and Western Blot analyses. Statistical analysis of data was performed by JAM, with the exception of the *in silico* analysis of enriched pathways, which was performed by CJM. JAM wrote the manuscript. All authors read and approved the final manuscript.

## Supplementary Material

Additional file 1 In silico prediction of omy-miRNA-122 regulated mRNA targets. Click here for file
